# Robotic-Assisted Surgery for Colorectal Cancer Treatment in 2026: An Updated Narrative Review

**DOI:** 10.3390/jcm15103714

**Published:** 2026-05-12

**Authors:** Cammarata Roberto, La Vaccara Vincenzo, Catamerò Alberto, Bani Lucrezia, Castagliuolo Pierpaolo, Giordano Federica, Castagna Vittoria, Coppola Roberto, Caputo Damiano

**Affiliations:** 1 Operative Research Unit of General Surgery, Fondazione Policlinico Universitario Campus Bio-Medico, 00128 Rome, Italy; 2 Department of Surgery, Campus Bio-Medico of Rome, 00128 Rome, Italy; 3 Medicine and Surgery, Sapienza University of Rome, 00185 Rome, Italy; 4 Medicine and Surgery, Campus Bio-Medico of Rome, 00128 Rome, Italy; 5 Research Unit of General Surgery, Università Campus Bio-Medico di Roma, 00128 Rome, Italy

**Keywords:** colorectal cancer, robotic-assisted surgery, commercially available robotic systems, robotic surgical platform, colectomy, robotic anterior resection, total mesorectal excision, da Vinci, Hugo RAS, Versius

## Abstract

**Background/Objectives**: Colorectal cancer (CRC) is one of the most commonly diagnosed malignancies worldwide and a leading cause of cancer-related mortality. Surgical resection remains the cornerstone of curative treatment. Over the past two decades, robotic-assisted surgery has emerged as an evolution of minimally invasive surgery, aiming to overcome several limitations of conventional laparoscopy. This narrative review summarizes the current state of the art of robotic surgery in CRC. **Methods**: A narrative review of the literature was conducted using PubMed/MEDLINE and Scopus databases, focusing on publications from 2015 to 2026. The review provides an overview of robotic platforms and summarizes the available clinical evidence. Priority was given to randomized controlled trials, meta-analyses, large observational studies, and clinical practice guidelines. The review focuses on major commercially available robotic systems, including the da Vinci^®^, Hugo™ RAS, and Versius^®^ platforms, as well as emerging robotic technologies. **Results**: Robotic colorectal surgery showed potentially favorable perioperative and oncological outcomes compared with laparoscopy. In rectal cancer, robotic approaches were associated with improved total mesorectal excision quality, lower conversion rates, and improved postoperative functional outcomes. Emerging evidence also suggested potential improvements in disease-free survival and local disease control following robotic rectal surgery. In colon cancer, robotic colectomy were associated with lower conversion rates, reduced blood loss, and faster postoperative recovery, with comparable long-term oncological outcomes. However, robotic procedures showed longer operative times and higher procedural costs. **Conclusions**: Robotic colorectal surgery appears to be a safe and effective minimally invasive approach, particularly in rectal cancer surgery. The development of new robotic platforms and increasing market competition may improve cost sustainability and expand its future role in colorectal cancer management.

## 1. Introduction

Colorectal cancer (CRC) is a malignant neoplasm affecting the colon or rectum. According to the latest GLOBOCAN estimates, CRC accounts for approximately 1.14 million new cases and 538,000 deaths annually worldwide. It ranks third in incidence and second in cancer-related mortality globally, with a higher incidence in older individuals and in males [1,2,3,4]. Rectal cancer is defined as a tumor located in the distal large bowel, up to 15 cm from the anal verge, as measured by rigid sigmoidoscopy. According to distance from the anal verge, rectal tumors are classified as low (≤5 cm), mid (>5–10 cm), or upper (>10–15 cm) [2]. The management of CRC is stage-dependent and requires a multidisciplinary approach integrating surgery and systemic therapies. According to current European Society for Medical Oncology (ESMO) Clinical Practice Guidelines, radical surgical resection is indicated for localized colon cancer (stages I–III) and for rectal cancer, either as primary therapy or after neoadjuvant treatment. In colon cancer, surgery aims to achieve complete tumor excision together with adequate regional lymphadenectomy. Retrieval of at least 12 lymph nodes is recommended to ensure accurate pathological staging and guide postoperative decisions on adjuvant chemotherapy. In rectal cancer, total mesorectal excision (TME) is the gold standard. It is critical to obtain negative circumferential and distal resection margins and to optimize local disease control. For locally advanced rectal tumors, neoadjuvant chemoradiotherapy is often employed to enhance resectability and oncological outcomes. For both colon and rectum cancer, adjuvant chemotherapy is tailored according to final pathological staging and tumor-specific risk factors [1,2,3,5].

While systemic therapies play an essential role in the multimodal management of colorectal cancer, surgical resection remains the only potentially curative treatment for localized disease. Chemotherapy and radiotherapy aim to reduce tumor burden, control micrometastatic disease, or improve resectability. Surgery provides definitive local disease control through complete tumor excision with adequate margins and lymphadenectomy. The surgical approach to CRC has evolved considerably over the past decades. Open surgery provides direct visualization and unrestricted instrument manipulation, but is associated with larger incisions, increased postoperative pain, higher wound complication rates, and prolonged recovery. The introduction of conventional laparoscopy in the 1990s represented a major advancement, offering reduced surgical trauma and faster recovery, while maintaining oncological equivalence. However, conventional laparoscopy presents inherent technical limitations, including two-dimensional visualization, a limited instrument range of motion, counterintuitive hand-eye coordination, tremor amplification, and ergonomic constraints that may contribute to surgeon fatigue. These limitations are relevant in technically demanding procedures such as TME, where precise dissection in the pelvic space is critical for oncological and functional outcomes.

Despite its key role in therapy, colorectal surgery is associated with non-negligible postoperative morbidity. The most common complications include ileus (11.8%), bleeding (7.6%), and surgical site infections (7.0%) [6]. Anastomotic leakage, one of the most dreaded complications, occurs in 2% to 19% cases [7]. Patients who suffer anastomotic leakage are at risk of end-organ dysfunction (33.3%), mortality (20.0%), reoperation (48.4%), and hospital readmission (20.6%) [6]. Furthermore, rectal resections can cause genitourinary and sexual dysfunction, due to the complexity of pelvic dissection and the proximity of autonomic nerves. Urinary dysfunction occurs in approximately one-third of patients [8,9]. One retrospective study found that 34.7% of patients develop erectile dysfunction and 29.8% develop ejaculatory dysfunction at 12 months after minimally invasive surgery [10].

Postoperative complications may also delay adjuvant treatment and negatively impact long-term oncological outcomes [11]. Starting adjuvant chemotherapy more than 6 weeks after surgery is associated with worse disease-free survival (hazard ratio 1.24) [12], while delays beyond 8 weeks are significantly associated with worse overall survival (HR: 1.37) [13].

Achieving optimal oncological radicality while minimizing perioperative complications is a priority in colorectal surgery. For these reasons, robotic-assisted surgery has emerged as a promising evolution. It may be particularly advantageous in rectal cancer surgery, obese patients, and the narrow male pelvis. Robotic surgery has addressed several intrinsic limitations of conventional laparoscopy, offering enhanced three-dimensional visualization, improved instrument dexterity, and tremor filtration.

In recent years, the evidence regarding robotic colorectal surgery has expanded, including randomized controlled trials, large observational studies, and multiple meta-analyses comparing robotic and laparoscopic approaches. At the same time, several new robotic platforms have entered clinical practice or are under development, contributing to a rapidly evolving technological landscape. However, no recent comprehensive narrative review has synthesized the current state of available robotic platforms with the latest clinical evidence. This review aims to provide an updated overview of the field, including robotic surgical systems, perioperative and oncological evidence, limitations, and future perspectives.

## 2. Methods

This narrative review was conducted to provide an updated overview of robotic surgery in colorectal cancer.

A literature search was performed using PubMed/MEDLINE and Scopus databases. The search focused on publications from January 2015 to February 2026. Seminal earlier studies were included when relevant for historical context. Search terms included combinations of “robotic surgery”, “robotic-assisted surgery”, “colorectal cancer”, “rectal cancer”, “colon cancer”, “colectomy”, “total mesorectal excision”, “da Vinci”, “Hugo RAS”, “Versius”, and “robotic platform”. Reference lists of included articles were also screened to identify additional relevant sources.

Studies were included if they met the following general criteria: (1) focus on robotic-assisted surgery for colorectal cancer in adult patients; (2) published in English in peer-reviewed journals; and (3) reported clinical, oncological, or technical outcomes relevant to the scope of this review. Studies were generally excluded if they meet the following criteria: (1) focused exclusively on benign colorectal disease without relevance to oncological principles; (2) single case reports, unless describing a novel technique or first-in-human application of a new platform; or (3) published in non-peer-reviewed sources, except for manufacturer documentation consulted for the technical specifications of robotic platforms.

Among eligible studies, priority was given to randomized controlled trials, systematic reviews and meta-analyses, large observational studies from national databases, and clinical practice guidelines from major scientific societies, including the European Society for Medical Oncology (ESMO) and the American Society of Colon and Rectal Surgeons (ASCRS). When multiple studies addressed the same topic, preference was given to the most recent, methodologically robust, or largest study.

As this is a narrative review, no formal systematic search protocol, risk of bias assessment, or quantitative data synthesis was performed. The aim was to provide a critical and clinically oriented synthesis of the literature rather than a systematic pooling of data.

## 3. Evolution of Robotic Surgery Through History

The historical development of robotics has been driven by both conceptual motivations and the practical needs of precision and safety. The term “robot” was coined in 1920 by the Czech writer Karel Čapek in Rossum’s Universal Robots. It derived from the word “robota” (labor) and originally referred to artificial beings designed to assist or replace human work [14].

Robots were first introduced into industrial production processes in the early 1960s, and their application in surgery began in the early 1980s. The first surgical robot used on a human patient was the PUMA 560 for neurosurgical biopsies in 1985. In 1988, the ProBot performed its first prostatic surgery, although its diffusion was limited due to technological limitations. Initial surgical robotic systems were based on teleoperation and rigid kinematic architectures. They were mainly applied in highly structured environments, such as orthopaedic and neurosurgical procedures. Examples include the ROBODOC and PUMA systems, where tasks primarily involved precise positioning and interaction with rigid anatomical structures [15,16]. These early experiences demonstrated the potential of robotic assistance for surgical precision. However, improvements in manipulation of deformable soft tissues were still required.

In the meantime, the widespread diffusion of minimally invasive surgery in the 1990s revealed the limitations of conventional laparoscopy. Instrument movement was counterintuitive due to the fulcrum effect, defined as the inversion of instrument motion caused by the fixed pivot point at the trocar site. Laparoscopy was also limited by reduced degrees of freedom, two-dimensional vision, a steep learning curve, and ergonomic constraints that affected surgical dexterity, particularly in demanding procedures. These limitations were especially relevant in colorectal surgery, where precise dissection, stable visualization, and fine instrument control are required [17,18,19]. Robotic surgery has emerged as an evolution of minimally invasive surgery, building upon the advantages of laparoscopy while addressing its technical limitations. The development of robotic platforms progressed through successive stages. Early systems focused on improving visualization and instrument control. AESOP (Automated Endoscopic System for Optimal Positioning) enabled surgeon-controlled endoscopic camera positioning, reducing dependence on a human assistant and representing a step toward increased autonomy and precision [16,20,21]. Subsequently, fully teleoperated robotic platforms were developed. During this phase, two competing systems emerged: the da Vinci Surgical System, developed by Intuitive Surgical, and the ZEUS robotic system, developed by Computer Motion based on the earlier AESOP system. Competition ended with the acquisition of ZEUS by Intuitive Surgical, which contributed to the establishment of the da Vinci system as the dominant platform [21,22].

Modern robotic platforms are designed to overcome the intrinsic limitations of conventional laparoscopy while preserving the benefits of minimally invasive surgery. They offer several key advantages. First, three-dimensional high-definition visualization delivers a magnified and immersive view of the surgical field. This surgeon-controlled, stable stereoscopic camera enables the precise identification of critical structures such as the autonomic nerve plexuses, ureters, and mesorectal fascia. Second, articulated robotic instruments (e.g., EndoWrist technology) provide seven or more degrees of freedom, compared with four degrees of freedom in conventional laparoscopy. Third, robotic software automatically compensates for the fulcrum effect, restoring intuitive hand-instrument concordance. Motion scaling translates the surgeon’s hand movements into finer instrument motions, while remor filtration eliminates physiological tremor. Fourth, the ergonomic console reduces physical strain and surgeon fatigue compared with prolonged laparoscopic procedures. Finally, emerging evidence suggests that the learning curve for robotic surgery may be shorter, particularly for complex procedures such as TME, potentially facilitating broader adoption. These technical features have supported the progressive adoption of robotic surgery in CRC, particularly for rectal resections, procedures in obese patients, and operations in patients with narrow pelvic anatomy. Over time, successive generations of the da Vinci platform have introduced refinements aimed at improving workflow integration, flexibility, and operative efficiency [22].

While the da Vinci system has continued to dominate the market, alternative platforms have emerged in recent years to address economic and logistical limitations. Systems such as the Versius Surgical System and the Hugo RAS System represent a new phase in surgical robotics. This phase is characterized by increased competition, modular design, and growing attention to cost sustainability. This technological diversification has further supported the expansion of robotic surgery across multiple surgical specialties, including urology, gynecology and general surgery [23,24,25].

## 4. Major Robotic Platforms in Colorectal Cancer Surgery

### 4.1. The da Vinci Multiport Robotic System

The da Vinci Surgical System (Intuitive Surgical, Sunnyvale, CA, USA) is the most widely adopted robotic platform in minimally invasive surgery. Introduced in the late 1990s and approved by the U.S. Food and Drug Administration (FDA) in 2000, the system pioneered robotic-assisted surgery and is the dominant robotic platform worldwide. Currently, more than 8600 da Vinci platforms are installed worldwide, including 1500 in Europe. The platform is based on a leader–follower architecture, in which the surgeon operates from a console while robotic arms reproduce the surgeon’s movements at the surgical site. This configuration allows the translation of hand movements into highly precise instrument motions while providing high-definition three-dimensional visualization, motion scaling, tremor filtration, and ergonomics [26,27].

#### 4.1.1. Evolution of the da Vinci Surgical Platform

Since its introduction, the da Vinci system has undergone continuous technological evolution through multiple generations. Early platforms, including the da Vinci Standard, S, and Si systems, progressively improved visualization, ergonomics, and instrument articulation. These technological developments enabled the widespread adoption of robotic surgery in urology, gynecology, thoracic surgery, and general surgery, including colorectal procedures [15]. The currently available multiport systems are the da Vinci Xi (IS4000) and da Vinci X (IS4200). Both platforms share a similar architecture consisting of three main components: the surgeon console, the patient cart, and the vision cart.

The surgeon console is a closed workstation with a 3D HD visualization and an intercom system. The console features ergonomic input systems and master manipulators for controlling the four robotic arms. Indocyanine green (ICG) fluorescence imaging is integrated into the system for intraoperative near-infrared visualization. The patient cart supports four robotic arms, called Universal Surgical Manipulators. These arms accommodate the endoscope and EndoWrist instruments. The latter provide seven degrees of freedom, exceeding the range of motion of the human hand and enabling complex intracorporeal maneuvers. The Xi platform introduced a rotating overhead boom that allows the robotic arms to be repositioned around the patient without moving the patient cart. This enables sequential access to multiple abdominal quadrants -such as the right colon, left colon, and pelvis- during a single procedure, without the need for redocking. These features are particularly advantageous in colorectal surgery, where multi-quadrant dissection is frequently required. The vision cart houses the image processing unit and provides video output for the surgical team. It integrates a dual-camera stereoscopic endoscope that delivers the three-dimensional image to the surgeon console and a two-dimensional display to the bedside assistants.

From an organizational perspective, the implementation of the da Vinci Surgical System requires dedicated instrumentation, including EndoWrist instruments (typically 10 uses per instrument), a stereoscopic endoscope, and compatible trocars. Structured training programs and technical support from the manufacturer are required [16,28,29].

In 2024, the da Vinci 5 system was introduced as the latest generation of the platform. While maintaining the multiport architecture, this system incorporates improved computing power, enhanced data integration, and the introduction of haptic feedback. This feature allows the surgeon to perceive forces applied by the robotic instruments, potentially improving tissue handling and surgical precision. Although clinical experience with this platform is still emerging, the introduction of haptic feedback represents a significant technological step in the evolution of robotic surgery [30,31].

The evolution of the da Vinci surgical platform is outlined in Table 1.

#### 4.1.2. Clinical Relevance in Colorectal Surgery

Over the past two decades, the widespread diffusion of the da Vinci platform has played a key role in the development of robotic colorectal surgery. Robotic assistance has been increasingly adopted for procedures such as colectomy and rectal resections, particularly in technically demanding pelvic dissections. Compared with conventional laparoscopy, the robotic platform offers improved visualization, dexterity, and ergonomics, which facilitate precise dissection in confined anatomical spaces such as the pelvis.

Despite these advantages, robotic surgery also presents several challenges, including the need for dedicated infrastructure, specialized training, and increased acquisition and maintenance costs. Nevertheless, the da Vinci platform remains the reference standard for robotic surgery worldwide, and its continuous technological evolution continues to shape the future development of robotic techniques in colorectal surgery [22,27,28].

### 4.2. The Hugo RAS Robotic System

The Hugo RAS system (Medtronic, Minneapolis, MN, USA) is one of the most recently introduced robotic platforms in minimally invasive surgery. In 2021, the first clinical procedures were performed in urology and gynecology in South America and Asia. In the same year, the system obtained CE marking for these indications, while in 2022, regulatory approval was extended to general surgery, enabling its gradual adoption in Europe [32,33]. Currently, the Hugo RAS platform is distributed in 25 countries worldwide. Over the past five years, 66 systems have been installed in Europe, 24 in Asia, 4 in Oceania, 1 in Canada, 2 in Africa, and 7 across Central and South America. In the United States, the platform remains experimental and is not yet commercially available. Compared with the da Vinci system, the adoption of the Hugo RAS platform is currently more limited [27,34].

The Hugo RAS system is a tele-operated platform with a modular architecture. It is composed of a surgeon console, four independent robotic arm carts, and a system tower integrating the laparoscopic column. This design allows each arm to be positioned individually around the operating table. This flexibility is particularly relevant in colorectal surgery, where multi-quadrant access and adjustable arm positioning are frequently required. However, this system has a longer learning curve for robot positioning and docking.

The surgeon console is an open workstation, with 3D HD visualization through dedicated glasses, hand controllers, and ergonomic adjustments. The four robotic arms provide eight degrees of freedom and can be configured flexibly, allowing the use of two to four arms depending on the procedure. The system tower can function as a standalone laparoscopic column. Individual robotic arms can also support the endoscope during laparoscopic procedures, enabling integration within existing workflows. From an organizational perspective, the implementation of the Hugo RAS platform requires dedicated instruments, optical systems, and electrosurgical equipment, as well as structured training protocols and technical support.

Overall, the Hugo RAS platform offers a modular design, differing from the centralized architecture of other systems and representing a key element in the comparison of emerging robotic platforms in colorectal surgery [27,35,36,37,38,39].

### 4.3. The Versius Robotic System

The Versius robotic system (CMR Surgical, Cambridge, UK) is a robotic platform designed for minimally invasive procedures. It has clinical utility across several specialties including general surgery, urology, gynecology, and thoracic surgery. The system was introduced into the European market in 2019 following CE certification, presenting an alternative to existing robotic surgical systems. Globally, approximately 174 Versius units are currently operational, reflecting the progressive adoption of the platform in several healthcare systems [27].

The Versius platform features a modular architecture with a surgeon console and four independent robotic arms mounted on mobile carts. Unlike some robotic systems, Versius does not include a proprietary laparoscopic tower. It is indeed designed to be compatible with standard laparoscopic equipment, including non-proprietary insufflators and other operating room devices. The open surgeon console provides 3D visualization through a passive display and allows operation in either seated or standing position. The Versius Plus model incorporates indocyanine green (ICG) fluorescence imaging. At the patient side, the system employs four independent robotic arms, one dedicated to the endoscope and three to surgical instruments. The instruments provide seven degrees of freedom and are interchangeable among the operative arms while maintaining compatibility with laparoscopic surgical workflows. From an organizational standpoint, as with other robotic platforms, the implementation of the Versius system requires dedicated robotic instruments, visualization systems, and electrosurgical equipment, as well as structured training programs for the surgical team.

In the context of colorectal surgery, the modular architecture and independent robotic arms may offer flexibility in operating room setup and port positioning. However, clinical adoption is still evolving, and its role in colorectal surgery remains to be defined [27,40,41,42].

Table 2 summarizes the key technical features of the major robotic surgical platforms.

## 5. Next-Generation Robotic Platforms

Beyond the robotic systems currently in widespread use, several emerging platforms are under development and may play an increasing role in colorectal surgery. Most have been developed as alternatives to the da Vinci Surgical System. Some are available only in selected countries, particularly in Asia, whereas others require further clinical data to define safety, feasibility, and potential indications in colorectal surgery. The growing number of platforms is increasing market competition and may improve economic sustainability. The following paragraphs provide a brief overview of these novel technologies.

### 5.1. da Vinci^®^ Single Port (SP)

The da Vinci Single Port (SP) system is designed for procedures performed through a single incision or natural orifice. Although it obtained CE marking in 2024 and began to be adopted in selected surgical fields, its role in colorectal surgery remains limited. Further studies are required to clarify its optimal indications [43].

### 5.2. Senhance^®^ Surgical System

The Senhance Surgical System (Asensus Surgical, Durham, NC, USA) is designed to support minimally invasive surgery using a laparoscopic-like interface. It has been introduced in several countries and applied in general surgery, gynecology, and urology. In colorectal surgery, early clinical experiences have demonstrated that Senhance-assisted procedures are feasible and safe, with perioperative outcomes comparable to conventional minimally invasive approaches. However, the current clinical evidence remains limited and largely based on single-center experiences and small observational studies. Although the system has been used for colectomy and rectal resections, its diffusion and clinical experience remain significantly lower compared with other robotic platforms. Therefore, its role in colorectal oncology is still under evaluation [44,45,46].

### 5.3. Hinotori™ Surgical Robot System

The hinotori Surgical Robot System (Medicaroid, Kobe, Japan) was approved for clinical use in 2022. It features independently mounted robotic arms with eight axes of motion, a 3D HD visualization system, and an ergonomic console [47]. A distinctive feature of the platform is the docking-free mechanism, which allows greater flexibility around the port site and may reduce excessive tissue traction during surgery [47,48].

Current evidence suggests that the hinotori represents a feasible and safe alternative for colorectal surgery, with short-term outcomes and oncological adequacy comparable to the da Vinci system [47,48,49,50]. However, propensity score-matched comparative analyses of hinotori-assisted rectal surgery have reported longer operative and console times compared with the da Vinci system [49,50]. These differences have been partly attributed to the limited availability of integrated surgical instruments, including advanced energy devices and stapling systems, during the early implementation phase [48].

To date, the hinotori Surgical Robot System is commercially available only in Japan, and further large-scale prospective studies are required to better define its long-term oncological outcomes and cost-effectiveness. Nevertheless, it represents an emerging platform that may potentially compete in the future global robotic surgery market [47,48,49,50].

### 5.4. Toumai^®^ Surgical Robot

The Toumai Surgical Robot (Shanghai MicroPort MedBot, Shanghai, China) is a next-generation platform with a modular architecture and advanced digital capabilities. It includes a 3D HD console, independently robotic arms, and an open platform compatible with different imaging systems and energy devices. This allows a flexible operating room setup and the potential integration of technologies such as tele-surgery and remote mentoring. From a health system perspective, emerging robotic platforms such as Toumai have been developed partly to address the economic barriers associated with conventional robotic surgery. Recent analyses highlighted these platforms aim to reduce overall procedural costs through reusable instruments, alternative procurement models, and potentially lower acquisition and maintenance costs. These economic considerations are particularly relevant in regions where cost has limited the adoption of robotic surgery [51]. Despite these promising technological and economic features, the current body of evidence on Toumai remains relatively limited, particularly in colorectal surgery. Most available data are derived from early clinical experiences or institutional reports. Robust comparative studies evaluating perioperative outcomes, oncological quality, and long-term results are still lacking. Therefore, further prospective investigations are necessary to clarify the clinical performance, safety profile, and cost-effectiveness.

### 5.5. Micro Hand S^®^ Robotic System

The Micro Hand S Surgical Robot (WEGO, Tianjin, China), developed in China, is a leader–follower platform designed as a cost-effective alternative to systems such as da Vinci. It uses two robotic arms controlled by the surgeon, while the 3D camera is operated by a human assistant, simplifying the setup and potentially reducing costs [51]. Available evidence indicates that it is safe and feasible for colorectal surgery, providing perioperative and oncological outcomes comparable to the da Vinci system. Despite longer operative and preparation times, it may represent a promising and potentially more accessible solution, particularly in healthcare systems seeking cost-efficient alternatives [51,52,53,54].

### 5.6. Revo-i^®^ Robotic Surgical System

The Revo-i Surgical Robot is a robotic platform developed in South Korea by Meere Company (Hwaseong, Korea). It includes a surgeon console, a patient cart with multiple robotic arms, and a 3D HD visualization system with articulated instruments and motion scaling. Like other next-generation systems, Revo-i has been introduced in the context of increasing global competition in robotic surgery, with the goal of improving cost-effectiveness while maintaining comparable clinical performance. Early clinical experience has been reported mainly in urologic surgery, particularly robot-assisted radical prostatectomy, demonstrating technical feasibility and acceptable perioperative outcomes. However, evidence regarding colorectal surgery remains limited, and further prospective studies are needed to assess surgical outcomes, oncological adequacy, and cost-effectiveness compared with established robotic systems [51].

## 6. Robotic Colorectal Cancer Surgery: Current Evidence

### 6.1. Robotic Anterior Resection (RAR) for Rectal Cancer

Evidence of robotic anterior resection for rectal cancer derives from multiple systematic reviews, meta-analyses, and randomized controlled trials. Overall, robotic rectal surgery has shown to be a feasible and generally safe minimally invasive approach. Growing evidence may suggests potential advantages over conventional laparoscopic surgery in several key domains, although the strength of evidence varies across outcomes and study designs.

#### Robotic Versus Laparoscopic Rectal Surgery

The comparison between robotic and laparoscopic rectal surgery has been evaluated in multiple randomized controlled trials with varying results. The ROLARR trial, the largest international multicenter RCT at the time (471 patients, 29 centers, 10 countries), found no significant difference in conversion to open surgery between robotic and laparoscopic groups (8.1% vs. 12.2%; adjusted OR 0.61, 95% CI 0.31–1.21, *p* = 0.16). No significant differences were observed in CRM positivity (5.1% vs. 6.3%), complication rates, or bladder and sexual function at 6 months [55]. Similarly, Kim et al. conducted a phase II RCT (139 patients) comparing robotic and laparoscopic surgery for rectal cancer and found comparable TME quality (80.3% vs. 78.1% complete TME), morbidity rates, and quality of life outcomes. However, robotic surgery demonstrated significantly better sexual function at 12 months postoperatively (*p* = 0.03) [56]. The COLRAR trial (295 patients) also found no significant difference in TME quality between robotic and laparoscopic surgery (80.7% vs. 77.1% complete TME), although a sub-analysis demonstrated significantly lower CRM positivity in the robotic group (0% vs. 6.1%, *p* = 0.031). Additionally, the robotic group had significantly shorter duration of opioid use (median 2 vs. 3 days, *p* = 0.001) [57]. The REAL randomized clinical trial (1171 patients) showed that robotic surgery achieved a lower 3-year locoregional recurrence rate (1.6% vs. 4.0%, HR 0.45, *p* = 0.03) and higher disease-free survival (87.2% vs. 83.4%, HR 0.74, *p* = 0.04) in middle and low rectal cancer [58]. This represents the first large, randomized trial to report significantly improved oncological outcomes with robotic surgery. However, it should be noted that the REAL trial required participating surgeons to have completed at least 50 robotic cases, which may limit the generalizability to less experienced centers.

Despite these limitations, real-world data from Germany (24,725 patients) and China (2702 matched patients) reported potential oncological advantages of robotic surgery, showing associations with improved overall survival and disease-free survival [59,60]. A multicenter propensity score-weighted cohort study from Japan (1053 patients, 16 hospitals) showed better 3-year relapse-free survival with robotic surgery (83.6% vs. 78.2%, HR 0.72, 95% CI 0.53–0.99) and a higher rate of pathological complete resection (98.2% vs. 95.3%) [61]. Moreover, a propensity score-matched analysis by Takamizawa et al., with 320 matched patients, showed lower postoperative complications with robotic surgery (Clavien–Dindo ≥II: 12% vs. 21%, *p* = 0.024) with comparable 5-year relapse-free survival (90.5% vs. 88.5%, *p* = 0.525) and overall survival (93.8% vs. 97.3%, *p* = 0.283) [62]. While these real-world studies are valuable, they remain susceptible to residual confounding, selection bias, and center-volume effects that propensity score methods cannot fully eliminate.

Robotic surgery has been associated with perioperative advantages over laparoscopy. Multiple meta-analyses have consistently reported a substantially lower risk of conversion to open surgery with the robotic approach. Khan et al., in a meta-analysis of 15 RCTs, reported a significantly lower conversion rate (RR 0.53, 95% CI 0.38–0.74, *p* = 0.0002), lower reoperation rate (RR 0.56, *p* = 0.03), higher TME completeness (RR 1.07, *p* = 0.03), and lower CRM positivity (RR 0.67, *p* = 0.01) [63]. The trial sequential meta-analysis by Pompeu et al. showed reduced conversion rates (RR 0.54, *p* = 0.002) and lower CRM positivity (RR 0.65, *p* = 0.017) with robotic surgery. However, Pompeu et al. noted that other outcomes remain underpowered and require further investigation [64]. Furthermore, a meta-analysis of 9 RCTs by Filho et al. reported reduced conversion rates, greater lymph node retrieval, and lower CRM positivity with robotic surgery, with comparable safety, mortality, and overall complication rates [65]. A meta-analysis of 11 RCTs showed that robotic surgery was associated with shorter hospital stay (*p* = 0.003), lower conversion rates (*p* = 0.0003), complication rates (*p* = 0.0009), blood loss (*p* = 0.007) and reoperation rates (*p* = 0.03), but longer operation time (*p* < 0.00001) [66]. In addition, robotic surgery has been associated with a lower rate of severe postoperative complications, particularly Clavien–Dindo (C-D) grade IV complications and anastomotic leaks [66,67].

Functional outcomes have been extensively investigated, with growing evidence suggesting potential improvements following robotic procedures. The REAL trial showed that robotic surgery provides significantly better urinary function at 3, 6, and 12 months postoperatively. It also enhanced male sexual function up to 12 months and female sexual function at 3 and 6 months. Improvements in defecation function were observed at 3 and 6 months [58]. However, these findings are not consistently supported across randomized evidence. The ROLARR trial found no significant differences in bladder or sexual function at 6 months [55], highlighting variability between studies. In contrast, meta-analyses showed that robotic surgery is associated with lower rates of postoperative urinary dysfunction and paralytic ileus [65,66,68,69,70,71]. Overall, these discrepancies indicate that the studies are heterogenous and functional outcomes may also be influenced by surgeon expertise and patient characteristics.

Despite these encouraging findings, the current evidence should be interpreted with caution. The heterogeneity across studies in terms of surgical experience requirements, patient selection criteria, tumor characteristics, and outcome definitions limits direct comparisons. Observational studies and national database analyses, while providing large sample sizes, are inherently subject to selection bias and unmeasured confounding. Moreover, the relatively short follow-up period of most available studies (3–5 years) limits definitive conclusions regarding long-term oncological outcomes. Further large-scale, multicenter randomized controlled trials with standardized outcome measures and extended follow-up are needed to confirm the potential oncological advantages of robotic rectal surgery.

### 6.2. Robotic Colectomy for Colon Surgery

Robotic colectomy outcomes have been evaluated in multiple systematic reviews, meta-analyses, and recent randomized controlled trials evaluating perioperative safety, oncological adequacy, and postoperative recovery. Across these studies, robotic colectomy appears to offer perioperative advantages with generally comparable oncological outcomes to laparoscopic techniques, although the quality of evidence varies across endpoints and most available data derive from observational studies and meta-analyses of heterogeneous trials.

#### Robotic Versus Laparoscopic Colectomy

Most available evidence focuses on the comparison between robotic and laparoscopic colectomy. Meta-analyses of randomized controlled trials consistently report a significantly lower conversion rate to open surgery with robotic procedures [65,66,72,73]. Large national database studies corroborate these findings, with conversion rates of 6.6% for robotic versus 11% for laparoscopic colectomy (*p* = 0.001) [73]. Robotic surgery is associated with reduced intraoperative blood loss in multiple studies, although the magnitude of this difference varies across analyses [66,73]. Operative time is consistently longer with robotic approaches, with mean differences ranging from 25 to 49 min depending on the procedure type [64,72,74].

From an oncological perspective, robotic surgery has been associated with several potential advantages. Meta-analyses report that robotic colectomy achieves greater lymph node retrieval [65,72,74] and lower rates of positive circumferential resection margins compared to laparoscopy [64,65]. However, these findings are largely derived from observational studies and small RCTs, and the clinical significance of these surrogate endpoints remains to be established. Overall survival and disease-free survival appear comparable between robotic and laparoscopic surgery in most available studies [64,75,76]. Despite this, few trials have been adequately powered to detect differences in survival endpoints.

Recent evidence suggests advantages in postoperative outcomes for robotic colectomy, though findings are not uniform across all endpoints. Large prospective cohort data from the National Surgical Quality Improvement Program (NSQIP) shows that robotic colectomy is associated with reduced postoperative morbidity (RR 0.84, *p* = 0.001), postoperative mortality (RR 0.83, *p* = 0.010), and postoperative ileus (RR 0.80, *p* = 0.001) compared to laparoscopy [77]. However, these NSQIP analyses are subject to selection bias, as robotic cases may be preferentially performed by higher-volume surgeons at higher-volume centers. Anastomotic leakage rates appear comparable between the two approaches in most analyses, although one large NSQIP study from Ahuja et al. reported higher anastomotic leak rates for left-sided robotic colectomy (3.4% vs. 2.7%, *p* = 0.001), warranting further investigation [78]. Recovery outcomes appear to favor robotic surgery over laparoscopy. A propensity-matched study reported shorter hospital stays (6.5 vs. 10.2 days, *p* = 0.005 for right colectomy; 5.5 vs. 8.2 days, *p* < 0.001 for left colectomy), lower rates of ileus and postoperative pain scores (3.0 vs. 4.1, *p* = 0.011 for right colectomy; 2.9 vs. 4.1, *p* < 0.001 for left colectomy) [79].

Time to first flatus and time to first stool are also significantly shorter after robotic surgery [64]. Multiple studies report shorter hospital stays with robotic surgery, with median differences of 1 day in large database studies [73,74]. Notably, a meta-regression analysis showed a temporal trend favoring robotic surgery, with complication rates improving over time as surgical experience increases (yearly change in Ln(RR): −0.062, *p* = 0.005) [80]. This suggests that the safety profile of robotic colectomy may continue to improve with growing adoption and surgeon experience. However, this temporal trend may also reflect concurrent improvements in patients’ selection, perioperative protocols and institutional learning rather than the robotic platform alone.

Finally, in obese patients, robotic surgery for both colon and rectal procedures has been associated with shorter hospital stay and lower readmission rates compared with laparoscopy [81,82].

Table 3 summarizes the key studies comparing robotic and laparoscopic approaches in colorectal surgery.

## 7. Limitations of Robotic Colorectal Surgery

Despite the rapid diffusion of robotic platforms in colorectal surgery, several limitations still restrict their widespread adoption. One of the most consistently reported drawbacks is the longer operative time associated with robotic procedures. In rectal cancer surgery, a systematic review comparing robotic and laparoscopic low anterior resection reported a mean increase of approximately 23 min in operative duration for the robotic approach [83]. Although this difference tends to decrease with increasing surgical experience, operative time remains an important factor influencing operating room efficiency and resource utilization.

Closely related to operative time is the impact of the learning curve on surgical outcomes. Available evidence suggests that proficiency in robotic colorectal surgery is generally achieved after 25–35 cases for individual procedures such as colectomy and TME, while establishing a comprehensive institutional robotic program may require approximately 60–75 cases [84,85,86].

Another major limitation is the economic burden associated with robotic surgery [87]. Multiple comparative analyses reported that robotic colorectal resections are associated with higher operative and total costs compared with laparoscopic procedures. This differential financial burden arises from the high capital investment, maintenance expenses, and the cost of dedicated instruments and disposable materials for robotic systems [88,89,90]. Available data indicate that robotic colectomy costs approximately $1300–3000 more per case than laparoscopic colectomy, with an increase of 14–25% depending on the procedure and healthcare setting [88]. When acquisition and maintenance costs are included, the total fixed cost has been estimated at approximately $1600 per procedure, in addition to variable costs related to longer operative times and dedicated consumables [89]. Cost-effectiveness analyses further support these findings. Bayesian meta-analyses of RCTs have suggested that laparoscopy represents the most cost-effective minimally invasive approach for colorectal surgery, combining shorter operative times with favorable perioperative outcomes [91]. Although robotic surgery may reduce intraoperative blood loss and conversion rates to open surgery, these advantages are often offset by the increased financial burden and longer operative duration [88].

## 8. Conclusions and Future Perspectives

In conclusion, current evidence suggests that robotic colorectal surgery may provide oncological and perioperative outcomes at least comparable to laparoscopy, while offering technical advantages, especially in rectal surgery. The REAL trial [58] suggest potentially favorable long-term oncological outcomes with the robotic approach, including lower locoregional recurrence and improved disease-free survival. Additionally, significant benefits in urinary, sexual, and defecatory function were reported. These findings are further supported by consistent evidence of reduced conversion rates and improved pathological quality indicators across multiple meta-analyses. Collectively, this evidence is consistent with an expanding role of robotic surgery in colorectal cancer management [63,64,65,66].

Despite these advances, several gaps in the current evidence warrant further investigation. First, additional large-scale, multicenter randomized controlled trials with extended follow-up (≥5 years) are needed to determine whether the oncological advantages of robotic surgery are confirmed across different patient populations and tumor stages. Second, most existing studies lack adequate patient stratification [92]; future research should include subgroup analyses based on tumor location (e.g., low vs. mid vs. high rectum), patient body mass index, pelvic anatomy (particularly the narrow male pelvis), and neoadjuvant treatment status to identify which patient populations derive the greatest benefit from the robotic approach. Third, there is a notable absence of standardized economic evaluations: single-center studies employ context-specific assumptions that limit generalizability [88]. Even systematic reviews are restricted by heterogeneous methodologies and the diversity of healthcare systems, making cross-institutional and international comparisons difficult [91]. Fourth, comparative data on emerging robotic platforms versus the established da Vinci system remain limited, with most evidence derived from early feasibility studies rather than head-to-head comparative trials [49,93,94,95,96]. Finally, the standardized reporting of functional outcomes using validated instruments (such as International Prostate Symptom Score (IPSS) for urinary function, International Index of Erectile Function (IIEF) for erectile function, and Low Anterior Resection Syndrome (LARS) score for bowel function) should be encouraged across future studies to enable meaningful cross-study comparisons.

The principal limitations of robotic surgery remain its higher costs and, in some settings, longer operative times. However, several strategies may contribute to overcoming these barriers. The increasing market competition from new robotic platforms is expected to drive down acquisition, maintenance, and consumable costs over time [23,25,51]. The development of reusable instruments and alternative procurement models, such as leasing arrangements and pay-per-use systems, may further reduce per-procedure expenses [51]. The robotic NICE (Natural Orifice IntraCorporeal Anastomosis with Extraction) procedure may reduce costs by avoiding abdominal wall incisions for specimen extraction and reducing wound-related complications [97,98]. Furthermore, high-volume robotic programs appear more cost-effective, likely due to lower complication rates and shorter hospital stays. This could suggest that centralization of robotic surgery in specialized centers may enhance economic sustainability [99,100,101,102].

Longer operative times remain a key barrier in expanding robotic access. Increasing surgical experience and the implementation of structured training programs, including simulation-based curricula and proctored case series, are associated with progressive decreases in operative duration [84,85,86,103]. The standardization of docking procedures, port placement protocols, and operating room setup can minimize non-operative time. While certain procedural steps intrinsic to robotic surgery, such as system docking, cannot be eliminated, they can be optimized through increasing technical proficiency and dedicated team training.

Looking ahead, the integration of artificial intelligence (AI) and machine learning technologies represents a promising frontier in robotic surgery. AI-based computer vision systems are being developed for real-time intraoperative tissue recognition, automated surgical phase identification, and ICG-based perfusion assessment. These technologies may improve surgical precision, reduce intraoperative complications, and support real-time decision-making [104,105,106]. Additionally, AI-enhanced simulation platforms and video-based performance assessment tools may accelerate the learning curve and improve training efficiency for robotic surgeons [106,107,108]. The first reported cases of real-time AI integration with robotic colorectal surgery have recently been published, demonstrating the technical feasibility of combining these technologies in clinical practice [109].

Another emerging frontier is telesurgery, which uses robotic platforms and high-speed, low-latency networks, such 5G wireless technology, to enable remote surgical assistance or fully remote procedures. Although still in its early stages, telesurgery may have the potential to expand access to specialized surgical expertise in underserved or geographically isolated areas. Recent systematic reviews have confirmed the technical feasibility of remote robotic surgery across multiple surgical specialties. However, significant challenges remain, including network stability, cybersecurity, regulatory frameworks, and costs [110,111]. As communication infrastructure continues to evolve, telesurgery may become an increasingly viable component of robotic surgical practice.

Overall, robotic surgery is poised to play an increasingly important role in the future landscape of minimally invasive colorectal surgery. As technological innovation continues, costs decrease, and clinical evidence matures, robotic platforms may become more widely accessible. In this evolving landscape, robotic surgery may eventually represent the standard of care for selected colorectal procedures, particularly in rectal cancer surgery.

## Figures and Tables

**Table 1:** Evolution of the da Vinci surgical platform.

Generation	Introduced in	Key Technological Features
da Vinci Standard	2000	First FDA-approved robotic surgical system
da Vinci S	2006	Improved ergonomics and visualization
da Vinci Si	2009	Dual console and improved imaging
da Vinci Xi	2014	Multi-quadrant access
da Vinci X	2017	Simplified configuration derived from Xi
da Vinci 5	2024	Haptic feedback

**Table 2:** Major robotic surgical platform.

Feature	da Vinci	Versius	Hugo RAS
Manufacturer	Intuitive Surgical	CMR Surgical	Medtronic
First clinical introduction	2000	2019	2021
System architecture	Centralized	Modular	Modular
Console type	Closed (immersive)	Open	Open
3D visualization	Integrated 3D viewer	Passive 3D display	Active 3D glasses
Fluorescence imaging	Yes	Yes (Plus model)	No
Number of robotic arms	4 (integrated)	4 (independent)	4 (independent)
Degrees of freedom	7	7	8
Haptic feedback	Yes (da Vinci 5 only)	No	No
Clinical diffusion	Adopted worldwide	Increasing global adoption	Early global adoption
Key features	Multi-quadrant access	Modular setup	Flexible multi-quadrant access

**Table 3:** Key findings from comparative studies: robotic vs. laparoscopic colorectal surgery.

Author/Year	Study Design	Tumor Location	Key Findings
Feng et al. 2025 [58]	RCT	Rectal (middle/low)	Lower 3-year locoregional recurrence; higher 3-year DFS; better urinary, sexual, and defecation function
Jayne et al. 2017 [55]	RCT	Rectal	No significant difference in conversion rate, CRM positivity, complications, or bladder/sexual function at 6 months
Park et al. 2023 [57]	RCT	Rectal (middle/low)	No significant difference in TME quality; lower CRM positivity in robotic sub-analysis
Kim et al. 2018 [56]	RCT	Rectal	Comparable TME quality, morbidity, and QoL; better sexual function at 12 months
Pompeu et al. 2025 [64]	Meta-analysis	Colorectal	Lower conversion rate and CRM positivity; longer operative time
Filho et al. 2025 [65]	Meta-analysis	Colorectal	Reduced conversion rates, greater lymph node retrieval, lower CRM positivity; comparable safety, mortality, and complication rates
Khan et al. 2024 [63]	Meta-analysis	Rectal	Lower conversion; lower reoperation; higher TME rate; lower CRM positivity
Huang et al. 2023 [66]	Meta-analysis	Colorectal	Lower conversion, complications, blood loss, reoperation; shorter hospital stay
Thrikandiyur et al. 2024 [80]	Meta-analysis	Colorectal	No overall difference in complications; significant temporal trend favoring robotic surgery (yearly Ln(RR) change: −0.062, *p* = 0.005); lower conversion
Wang et al. 2020 [67]	Meta-analysis	Rectal	Lower severe complications; lower C-D grade IV; lower anastomotic leak
Negrut et al. 2024 [74]	Meta-analysis	Colon	Longer operative times, shorter hospital stay; lower conversion; higher lymph node retrieval; longer operative time
Piso et al. 2026 [59]	Observational	Rectal	Higher OS; lower CLR and conversion
Mizuno et al. 2025 [61]	Observational	Rectal (middle/low)	Higher 3-year RFS; higher complete resection rate; comparable complications
Takamizawa et al. 2025 [62]	Observational	Rectal	Lower C-D ≥II complications; lower ileus; comparable 5-year RFS and OS
de Almeida Leite et al. 2024 [77]	Observational	Colon	Lower morbidity; lower mortality; longer operative time
Emile et al. 2023 [73]	Observational	Colon	Lower conversion; shorter hospital stay; comparable 30-day and 90-day mortality

Abbreviations: RCT, randomized controlled trial; TME, total mesorectal excision; CRM, circumferential resection margin; DFS, disease-free survival; OS, overall survival; RFS, relapse-free survival; CLR, cumulative locoregional recurrence; C-D, Clavien–Dindo; QoL, quality of life.

## Data Availability

No new data were created; data derive from current evidence.
